# Comparative Neuroprotective Evaluation of Mitochondria-Targeted Antioxidant MitoQ Versus Acetyl-L-Carnitine in Vincristine-Induced Peripheral Neuropathy: Electrophysiological and Structural Insights

**DOI:** 10.3390/antiox15101308

**Published:** 2026-10-10

**Authors:** Ahmet Akkoca, Burcu Gültekin

**Affiliations:** 1 Department of Biophysics, Faculty of Medicine, Necmettin Erbakan University, Konya 42090, Türkiye; 2 Department of Histology and Embryology, Faculty of Medicine, Necmettin Erbakan University, Konya 42090, Türkiye; bgultekin@erbakan.edu.tr

**Keywords:** acetyl-L-carnitine, compound action potential, MitoQ, peripheral neuropathy, sciatic nerve, vincristine

## Abstract

Vincristine (Vin), a chemotherapeutic agent commonly used in clinical practice, frequently causes peripheral neuropathy (PN). The aim of this study was to compare the potential therapeutic effects of acetyl-L-carnitine (ALC) and the mitochondria-targeted antioxidant MitoQ, administered following the establishment of PN in rats. PN was induced in rats by administering Vin (0.1 mg/kg/day) for 5 consecutive days, followed by a 2-day break, and then for another 5 days (cumulative dose: 1 mg/kg). Animals were subsequently treated daily for 7 days with either 300 mg/kg/day of ALC or 2 mg/kg/day of MitoQ. At the end of the study, sciatic nerve function was assessed using ex vivo electrophysiological recordings, while morphological changes were examined via H&E, Klüver–Barrera, and MBP/MPZ immunohistochemical staining. Vin exposure caused significant impairments in sciatic nerve CAP amplitude, integral, and conduction velocity, and histopathologically led to myelin damage, axonal degeneration, and vacuolization. Both treatments administered after the injury significantly attenuated functional and structural impairments, restoring them toward baseline levels. Notably, MitoQ showed favorable numerical improvements across myelin sheath integrity, vacuolization scores, and CAP parameters. In conclusion, MitoQ demonstrates notable neuroprotective efficacy and promotes nerve structural recovery following Vin-induced PN.

## 1. Introduction

Peripheral neuropathy (PN) is a common pathological condition resulting from trauma, systemic diseases, or toxic exposure, leading to substantial sensory, motor, and autonomic dysfunction. Studies on this topic indicate that the prevalence of PN ranges from 1% to 7% of the population, depending on the underlying causes [1]. Chemotherapy agents are among the primary causes of PN. Although the literature reports varying results, recent studies have found the prevalence of PN in cancer patients receiving chemotherapy to be >50% [2,3]. This high rate underscores the importance of research into the prevention or treatment of chemotherapy-induced PN.

Vincristine (Vin) is a vinca alkaloid derived from *Catharanthus roseus* that exerts its anticancer effect by disrupting the stabilization of microtubule filaments and arresting the cell cycle in the G2/M phase. Due to its high systemic toxicity, Vin is generally administered in combination protocols for various cancer therapies [4]. Among its broad spectrum of adverse effects, peripheral neuropathy (PN) is particularly prominent and often serves as a dose-limiting factor due to severe neuropathic pain [5]. Indeed, treatment-induced PN can lead to long-term disability, with pathological symptoms persisting well beyond the cessation of chemotherapy [6]. Various cellular and experimental animal models have been developed to elucidate the underlying molecular mechanisms of this condition and to develop new treatment strategies. Accumulating literature suggests that oxidative stress, metabolic disturbances, and secondary mitochondrial impairment are implicated in the pathogenesis of Vin-induced neurotoxicity [7]. Specifically, vincristine disrupts the mitochondrial membrane potential and impairs respiratory chain complex activities (particularly complexes I and IV). This failure induces mitochondrial calcium (Ca^2+^) overload, excessive superoxide generation, and premature opening of the mitochondrial permeability transition pore (mPTP). Consequently, the resulting depletion of cellular ATP compromises kinesin-mediated axonal transport, disrupts mitochondrial fission-fusion dynamics, and ultimately triggers the release of cytochrome c to initiate axonal degeneration [8,9]. The development of an effective therapeutic approach will not only facilitate the safe administration of chemotherapy but also contribute to improving patients’ quality of life and functional recovery during the post-treatment period [10].

The sciatic nerve is the primary motor and sensory nerve of the lower extremity. It performs essential functions such as walking, knee flexion, foot movement, and sensory transmission [11]. However, chemotherapeutic agents commonly used in clinical practice can cause significant structural and functional damage to the sciatic nerve. In experimental studies on Vin-induced sciatic nerve damage, axonal swelling, damage to the myelin sheath, Schwann cell damage, and a decrease in fiber density are among the common findings [12,13]. Many studies point to increased oxidative stress, lipid peroxidation, and myelin damage as the causes of these pathologies induced by Vin [14]. Consequently, investigating targeted antioxidant strategies to counteract Vin-induced neurotoxicity is of critical importance.

Acetyl-L-carnitine (ALC) is an ester derivative of L-carnitine, an endogenous molecule that plays critical roles in lipid metabolism. Its most important functions include transporting long-chain fatty acids into the mitochondria for beta-oxidation and contributing to ATP production. Exogenously administered ALC is known to exert potent therapeutic effects through its antioxidant properties [15]. Prior studies have demonstrated the pronounced neuroprotective efficacy of ALC in peripheral neuropathies of diverse etiologies. In experimental models of diabetic, antiretroviral, and chemotherapy-induced neuropathies (such as paclitaxel and cisplatin), ALC administration has been shown to alleviate mechanical allodynia, enhance nerve conduction velocity, and promote axonal regeneration. At the cellular level, these protective actions are mediated through the restoration of acetyl-CoA pools, up-regulation of nerve growth factor (NGF) and its high-affinity receptor TrkA, and attenuation of non-enzymatic protein glycation and lipid peroxidation in peripheral nerves [16,17,18]. On the other hand, damage to the mitochondrial electron transport chain due to various causes leads to the excessive production of reactive oxygen species (ROS), making the mitochondria a major source of oxidative stress. Critically, the primary targets of excessive mitochondrial ROS are the mitochondria themselves, triggering a vicious cycle of bioenergetic failure and metabolic impairment [19]. Over the past twenty-five years, antioxidant molecules have been targeted toward the mitochondria—the primary source of oxidative damage—via a lipophilic cation commonly known as triphenylphosphonium (TPP^+^) [20]. Thanks to this strategy, ROS produced in the mitochondria are eliminated without causing damage to the mitochondria—their primary target—and before they enter the circulatory system and reach other tissues. MitoQ is the most widely used molecule and was developed by Kelso et al. (2001) as the first mitochondria-targeted antioxidant through the covalent binding of coenzyme Q to TPP^+^ [21]. Beyond its potent free radical scavenging capacity, MitoQ exhibits well-documented anti-apoptotic and anti-inflammatory properties. In experimental models of neurotoxicity and nerve injury, MitoQ has been reported to directly suppress intramitochondrial lipid peroxidation, preserve mitochondrial cristae morphology, and prevent structural axonal degeneration. While ALC acts ubiquitously across cellular compartments primarily by replenishing energy substrates and buffering systemic oxidative stress, MitoQ provides site-specific protection by neutralizing reactive species directly within the mitochondrial matrix [22,23]. Consequently, comparing this organelle-targeted approach against the broader metabolic profile of ALC establishes a clear comparative framework for assessing neuroprotective efficacy against vincristine-induced nerve damage.

In this study, the neuroprotective efficacy of ALC, a conventional antioxidant, and MitoQ, a mitochondria-targeted antioxidant, was comparatively evaluated in a Vin-induced peripheral neuropathy model. The primary objective of the study was to electrophysiologically demonstrate the effects of Vin administration on sciatic nerve compound action potential (CAP) parameters and to compare the efficacy of two different antioxidant strategies in improving nerve conduction against this damage. Changes in nerve conduction were evaluated not only through standard CAP parameters but also via nerve conduction velocity distribution (NCVD) analysis, with the aim of providing a more detailed assessment at the level of different axon populations. In addition, structural changes in nerve tissue were assessed through histological and immunohistochemical analyses to identify morphological differences accompanying the electrophysiological findings. Thus, the comparative neuroprotective profile of a mitochondria-targeted antioxidant strategy versus conventional antioxidant therapy was systematically characterized in Vin-induced neurotoxicity.

## 2. Materials and Methods

### 2.1. Experimental Animals and Groups

To avoid sex-dependent variations in baseline electrophysiological properties and ensure experimental homogeneity, 42 adult female Wistar-Albino rats (8–10 weeks of age; initial body weight: 336.55 ± 30.94 g, mean ± SD) were used. Inclusion criteria specified healthy adult female rats exhibiting normal baseline physical activity and an appropriate body weight range. No predefined data exclusion or outlier criteria were applied, and no animals or data points were excluded from the study (final sample size: *n* = 7 per group across all analyses). Body weight was systematically recorded for each animal using a calibrated digital balance at baseline (Day 1) and prior to sacrifice (Day 20) to monitor overall health status and toxicity. All experimental procedures were approved by the Local Ethics Committee for Animal Experiments at Necmettin Erbakan University (approval no.: 2025-096, 30 October 2025). All stages of the study were conducted under the supervision of officials from the Necmettin Erbakan University Center for Experimental Medicine, Application, and Research to ensure animal welfare. All rats were randomly assigned to 6 experimental groups (*n* = 7 per group) using a computer-generated random number sequence, while ensuring baseline body weight balance across groups. The animals were housed in group-labeled polypropylene cages under strictly standardized environmental conditions (temperature: 22 ± 2 °C, relative humidity: 50–60%, and a 12 h light/dark cycle) with identical rack positioning and ad libitum access to standard rat chow and water. The sample size of *n* = 7 per group was determined based on a priori power estimation using CAP amplitude as the primary outcome parameter. Based on historical variance in rat sciatic nerve electrophysiology, a sample size of 7 animals per group provides greater than 80% statistical power (alpha = 0.05) to detect meaningful biological differences among control, neurotoxicity, and treatment groups.

Control group (Con, *n* = 7): Intraperitoneal (i.p.) injections of physiological saline (PS) were administered in the same volume as the 10-day Vin and 7-day antioxidant treatments, serving as the solvent for a total of 17 days.

Vincristine group (Vin, *n* = 7): Vin (T1270, Targetmol, Shanghai, China) was dissolved in PS and prepared fresh daily. The Vin-induced PN model was established, as previously described in the literature, by administering 0.1 mg/kg/day i.p. injections for 5 consecutive days, followed by a 2-day break, and then another 5 consecutive days of Vin i.p. injections [24]. Subsequently, PS (vehicle) was administered via i.p. injection in an equivalent volume for 7 consecutive days, in parallel with the antioxidant treatment schedules.

Acetyl-L-carnitine group (ALC, *n* = 7): ALC (BD135753, BLDpharm, Shanghai, China) was dissolved in PS and prepared fresh daily. In parallel with the 10-day Vin treatment, PS was administered via injection, followed by i.p. injections of 300 mg/kg/day ALC for 7 days, which is known to improve mitochondrial bioenergetics [25].

MitoQ group (*n* = 7): MitoQ (T12059L, Targetmol, Shanghai, China) was dissolved in PS, aliquoted into daily doses, and stored at −20 °C. PS injections were administered in parallel with the 10-day Vin regimen, followed by 7 days of MitoQ administration at 2 mg/kg/day via i.p. injection [26].

Vincristine + Acetyl-L-Carnitine group (Vin+ALC, *n* = 7): Following a 10-day course of Vin (0.1 mg/kg/day) as described in the literature, animals in this group received i.p. injections of ALC at a dose of 300 mg/kg/day for 7 days.

Vincristine + MitoQ group (Vin+MitoQ, *n* = 7): Animals in this group received a 10-day course of Vin (0.1 mg/kg/day) as described in the literature, followed by 7 days of MitoQ administered via i.p. injection at a dose of 2 mg/kg/day.

**Figure 1:**
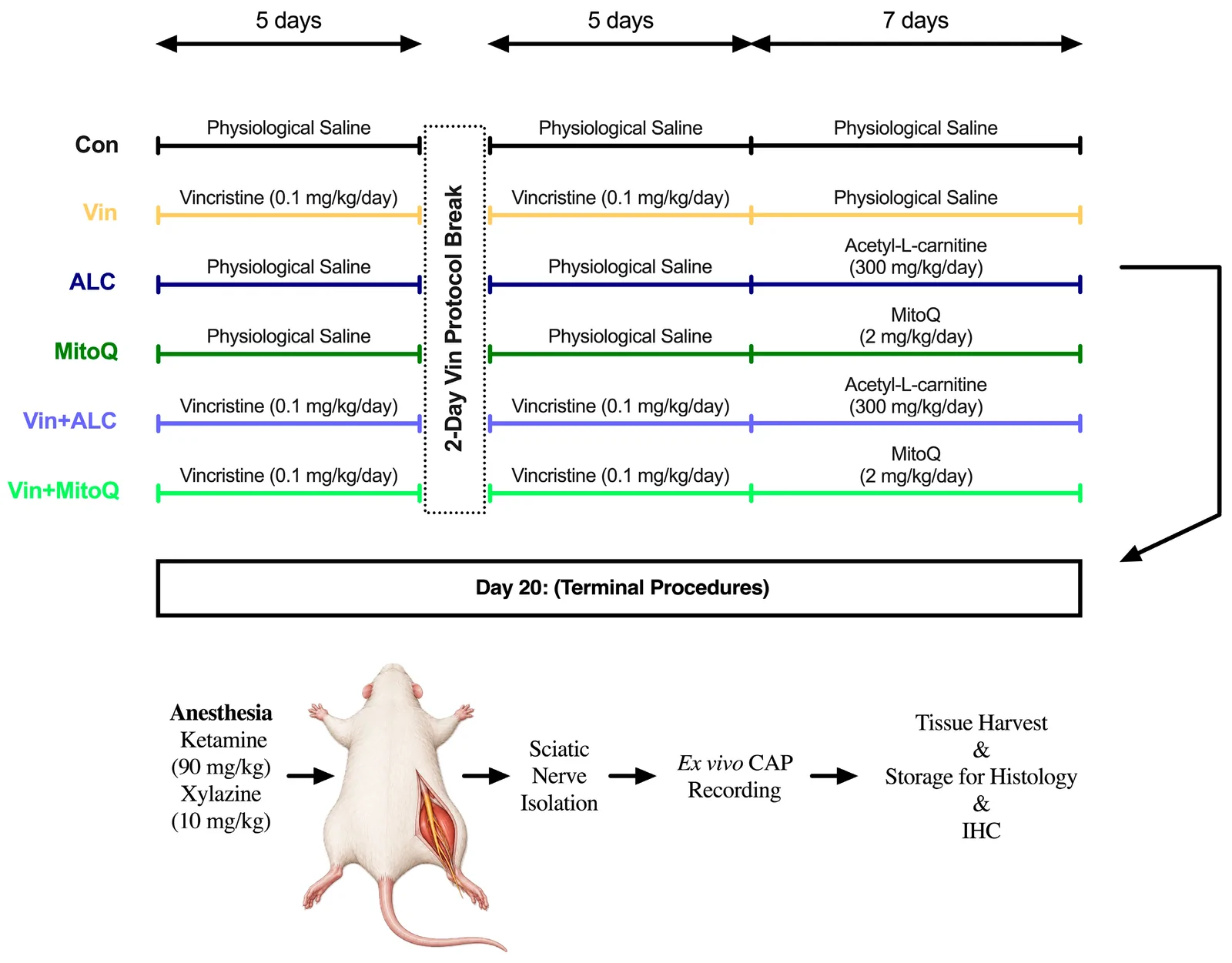
Schematic timeline of the experimental design and procedures.

At the end of the injection procedures, all rats were anesthetized with a combination of 90 mg/kg ketamine and 10 mg/kg xylazine and then sacrificed by cervical dislocation. Following sacrifice, CAP electrophysiological recordings were obtained from the rats’ right sciatic nerves. The left sciatic nerves were stored for histological and immunohistochemical experiments. All of these procedures are schematically shown in Figure 1.

### 2.2. Compound Action Potential (CAP) Recordings and Analysis

Right sciatic nerves were rapidly isolated from the animals and placed in freshly prepared Krebs solution. Krebs solution (mmol/L): NaCl 119, KCl 4.8, CaCl_2_ 1.8, MgSO_4_ 1.2, KH_2_PO_4_ 1.2, NaHCO_3_ 20, and glucose 10. The solution was continuously gassed with a mixture of 95% O_2_ and 5% CO_2_ to maintain physiological pH and oxygen levels. CAP recordings from isolated sciatic nerves in a steel-pinned organ bath were digitized using the BSLCBL4B cable set and Biopac MP36 systems. To trigger CAP generation, supramaximal square wave stimuli were delivered to the sciatic nerves via the Grass S48 stimulator and the Grass SIU5 stimulus isolation unit. The distance between the recording electrode and the stimulus electrode was kept constant at 3 cm. Using this distance, the maximum conduction velocity (V_max_, m/s) was calculated based on the time interval between the onset of the stimulus artifact and the onset of the CAP. Additionally, the CAP integral (representing total electrical activity), peak amplitude, and maximum rates of voltage change during the depolarization and repolarization phases (+dV/dt_max_ and −dV/dt_max_) were determined [27].

In addition, an NCVD analysis was performed using raw data obtained from CAP recordings. The relative contributions of nerve fiber groups with different conduction velocities to the CAP were calculated using a modified model developed by Tuncer et al. (2011), which is based on the advanced mathematical model by Cummins et al. (1979) [28,29]. This method allows for the separate identification of the individual contributions of axon populations of different diameters within a nerve to the conduction velocity, rather than relying on a single conduction velocity value. Thus, this approach enabled the differential characterization of specific fiber sub-populations (fast-conducting large-diameter versus slow-conducting thin fibers) most vulnerable to injury or responsive to antioxidant interventions. To enhance visual clarity, the NCVD results were externally visualized by categorizing them into different speed groups: slow (<25 m/s), medium (25–45 m/s), and fast (>45 m/s) [30]. To prevent experimental bias, ex vivo sciatic nerve isolations, CAP electrophysiological signal acquisitions, and subsequent NCVD mathematical calculations were performed by investigators blinded to the experimental treatment codes of the animals.

### 2.3. Tissue Processing and Slide Preparation

Rapidly isolated sciatic nerve tissues were fixed in 10% neutral buffered formalin for 48 h. Following fixation, the tissues were dehydrated using a series of increasing alcohol concentrations, cleared with xylene, and embedded in paraffin blocks after routine tissue processing procedures. Transverse sections (2 µm thick) were cut from the paraffin blocks using a rotary microtome and mounted onto positively charged slides.

### 2.4. Hematoxylin and Eosin Staining (H&E)

Hematoxylin–eosin (H&E) staining was applied to the sections to evaluate general nerve morphology. The tissue sections were deparaffinized in xylene twice, for 15 min each time, and then dehydrated in 100% (twice), 95%, 80%, and 70% alcohol for 3 min each. The nuclei of the tissues were stained with hematoxylin for 4 min, followed by staining with eosin for 40 s to stain the extracellular matrix and cytoplasm. The sections were dehydrated and mounted with synthetic tissue mounting medium (1.07961, Sigma-Aldrich, St. Louis, MO, USA).

### 2.5. Klüver–Barrera (KB) Staining (Luxol Fast Blue + Cresyl Violet) and Scoring

Sciatic nerve sections were stained using the Luxol Fast Blue (LFB) staining kit (PLKit345-150, Patolab Biomedical, İstanbul, Türkiye) according to the manufacturer’s recommended protocol to assess myelin integrity. After deparaffinization and rehydration, the sections were incubated in a 0.1% LFB solution at 56–60 °C. A lithium carbonate solution was used during the differentiation step, and contrast staining was performed with Cresyl Violet. Following dehydration and clearing, the sections were mounted with tissue mounting medium.

The histological analysis of sciatic nerve tissue sections stained with H&E and KB was based on the evaluation of four different parameters, including myelin sheath damage, axonal degeneration, vacuolization, and structural irregularities in nerve fibers. Histopathological scoring was blindly performed across five randomly selected, non-overlapping fields per specimen (40× magnification) by grading lesion severity on a 4-point scale: 0 = absent/normal, 1 = mild (occasional focal lesions), 2 = moderate (multifocal distinct changes), and 3 = severe (diffuse, widespread structural damage) [31]. All histopathological evaluations were performed using a blinded approach with a Zeiss Lab. A1 light microscope (Carl Zeiss, Jena, Germany)and a Zeiss AxioCam ERc 5s camera imaging system (ZEN Lite software, version 3.1, Carl Zeiss, Jena, Germany); tissue slides were assigned randomized code identifiers by an independent researcher, ensuring that the evaluating histopathologists were completely unaware of the experimental group allocations during microscopic scoring and image analysis.

### 2.6. Immunohistochemical Staining and Scoring

Then, 2 μm thick sections of sciatic nerve tissue were mounted on lysine-coated slides. The slides were incubated in xylene for 30 min and then subjected to a 20 min heat-mediated antigen retrieval procedure in citrate buffer (pH 6.0). A 3% hydrogen peroxide (H_2_O_2_) solution was used to suppress endogenous peroxidase activity. Following antigen retrieval, the sections were incubated with a ready-to-use Serum Blocking Buffer (E-IR-R217A; Elabscience Biotechnology Inc., Wuhan, China) to prevent nonspecific binding. Tissue sections were incubated overnight at 4 °C with anti-MBP (10458-1-AP, 1:200; Proteintech Group, Rosemont, IL, USA) and anti-MPZ (10572-1-AP, 1:200; Proteintech Group, Rosemont, IL, USA) primary polyclonal antibodies. Following the primary antibody incubation, the sections were incubated with an IgG secondary antibody (SA00001-2, Proteintech Group, Rosemont, IL, USA) at a 1:200 dilution ratio at room temperature for 30 min. To visualize the immunological reaction, a 3,3′-diaminobenzidine (DAB) substrate and chromogen kit (PL-060-HD, PatoLab Biomedical, İstanbul, Türkiye) was used, and Mayer’s hematoxylin was used for counterstaining. For negative controls, PBS was incubated in place of the primary antibodies. The sections were evaluated using a Zeiss Lab. A1 light microscope, and photographs were taken using the Zeiss Axicam ERc 5s camera imaging system.

For MBP quantification, five randomly selected high-magnification fields per sciatic nerve section were analyzed. After color deconvolution using ImageJ 1.54p, the OTSU threshold algorithm was applied. Results were presented as the percentage of total area stained [32].

### 2.7. Statistical Analysis

NCVD analyses were performed using MATLAB R2026a, while statistical analysis of other data was conducted using GraphPad Prism 10.3.1 software. The normality of the data was tested using the Shapiro–Wilk test. Longitudinal body weight changes measured at baseline and terminal timepoints were analyzed using repeated-measures two-way analysis of variance (RM Two-Way ANOVA) with time as the within-subject factor and treatment group as the between-subject factor, followed by Sidak’s post hoc test. Other continuous endpoints following a normal distribution were analyzed using one-way ANOVA followed by a post hoc Tukey test. Data are presented as mean ± standard deviation. Since semi-quantitative histological scores are ordinal data and do not require a normal distribution, they were analyzed using the Kruskal–Wallis test followed by the Dunn post hoc test. These data are presented as median (Q1–Q3). In statistical analyses, *p* < 0.05 was considered statistically significant. Additionally, to evaluate the magnitude of treatment effects beyond binary *p*-value thresholds, effect sizes (partial eta-squared for ANOVA and partial epsilon-squared for Kruskal–Wallis) and 95% confidence intervals were evaluated across key parameters. Additionally, to ensure complete data transparency and visualize individual biological variability across small sample sizes (*n* = 7/group), quantitative bar charts have been updated to include superimposed individual data points (dot plots), with error bars consistently presented as Mean ± SD.

## 3. Results

The neuroprotective efficacy of conventional antioxidant (ALC) and mitochondria-targeted antioxidant (MitoQ) therapies against peripheral neuropathy induced by Vin was investigated through electrophysiological and histological evaluations.

Initial and final body weights were recorded for all experimental groups. Over the experimental period, percentage body weight changes across groups were −11.4% (345.3 ± 39.3 g to 306.0 ± 37.5 g) for Con, −15.0% (346.7 ± 46.1 g to 294.7 ± 48.1 g) for Vin, −4.7% (338.3 ± 37.5 g to 322.3 ± 41.0 g) for ALC, −6.5% (333.6 ± 27.8 g to 311.9 ± 34.8 g) for MitoQ, −9.7% (323.6 ± 9.3 g to 292.3 ± 10.4 g) for Vin+ALC, and −12.2% (331.9 ± 13.0 g to 291.4 ± 19.1 g) for Vin+MitoQ. These percentage reductions in body weight did not show any statistically significant difference among the experimental groups, with repeated-measures two-way ANOVA revealing no significant interaction between time and treatment group, nor any significant main effect of treatment (*p* > 0.05).

**Figure 2:**
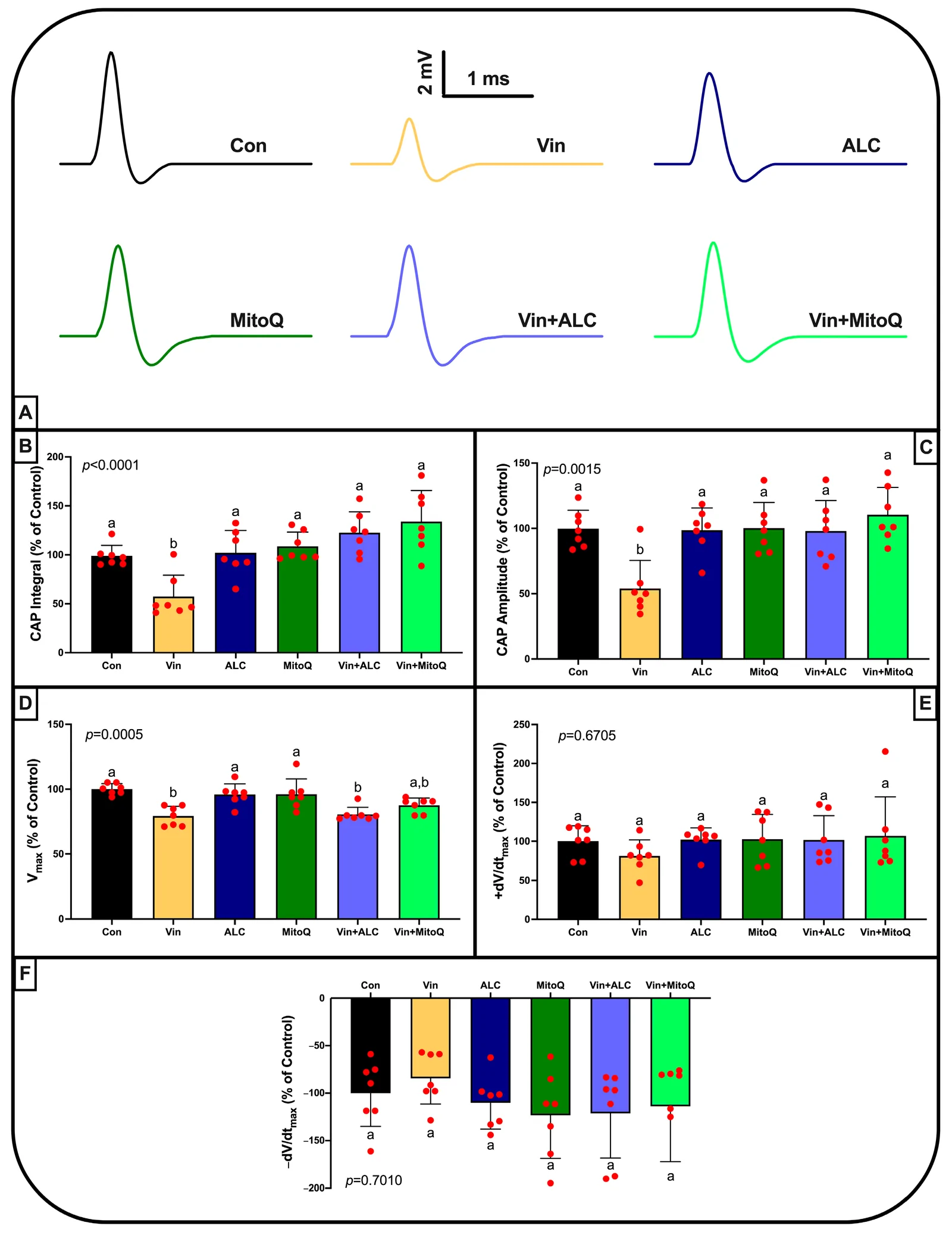
Electrophysiological analysis of compound action potential (CAP) data recorded from an isolated sciatic nerve. (**A**) Sample CAP waveforms from all experimental groups (scale bars: 2 mV and 1 ms). (**B**–**F**) Quantitative CAP parameters presented as a percentage of the control group (% of control): (**B**) CAP integral, (**C**) CAP amplitude, (**D**) maximum nerve conduction velocity (V_max_), (**E**) maximum rise rate (+dV/dt_max_), and (**F**) maximum fall rate (−dV/dt_max_). Statistical analyses were performed using one-way analysis of variance (One-Way ANOVA) followed by a post hoc Tukey test. Data are presented as mean ± standard deviation (mean ± SD). Groups sharing the same letter indicate no statistically significant difference, whereas groups labeled with different letters indicate a statistically significant difference (*p* < 0.05), based on the post hoc test results.

The physiological activity of the sciatic nerve was assessed using CAP parameters obtained from isolated sciatic nerves. Vin exposure resulted in significant reductions in CAP integral, amplitude, and Vmax values. Administration of ALC or MitoQ alone to healthy animals produced no adverse functional changes. Importantly, therapeutic administration of either antioxidant significantly restored CAP integral and amplitude compared to the Vin group. Regarding the V_max_ parameter, ALC treatment failed to reverse the vincristine-induced decline (remaining statistically comparable to the Vin group). In contrast, the Vin+MitoQ group exhibited an intermediate recovery profile, as its conduction velocity did not differ significantly from either the healthy control or the Vin group (bearing both ‘a’ and ‘b’ notations). Thus, while MitoQ exhibited a numerical trend closer to baseline levels for maximum conduction velocity, no statistically significant difference was observed between the Vin+MitoQ and Vin+ALC cohorts (Figure 2A–D). On the other hand, there were no statistically significant differences between the groups in either the +dV/dt_max_ or −dV/dt_max_ parameters (Figure 2E,F).

**Figure 3:**
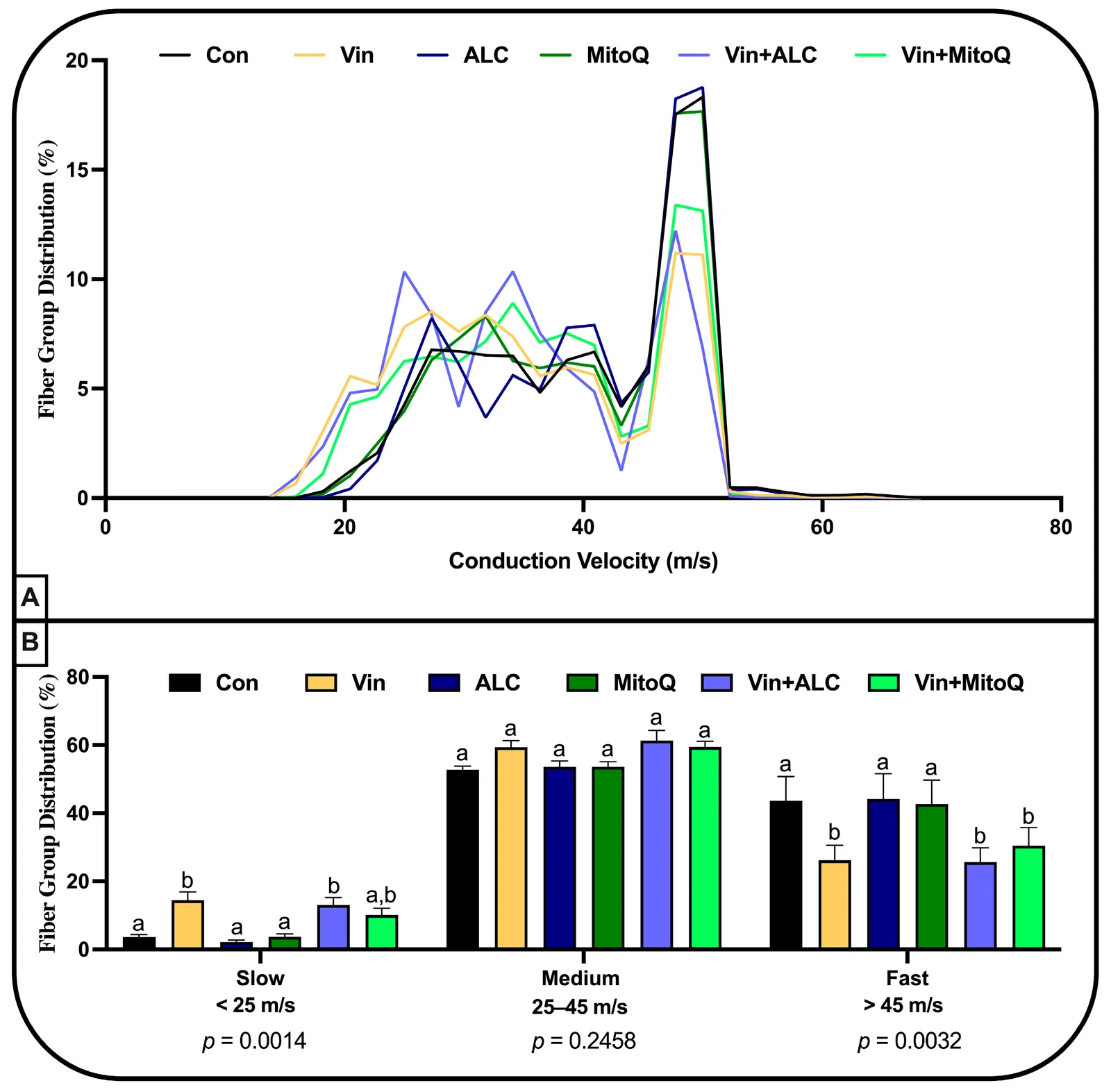
Distribution of nerve conduction velocities (NCVD) in isolated sciatic nerve fibers. (**A**) Graph showing the continuous distribution percentages of nerve fiber groups at different conduction velocities. (**B**) Quantitative comparison of the relative percentages of fiber groups categorized according to three different conduction velocity classifications: Slow (<25 m/s), Medium (25–45 m/s), and Fast (>45 m/s) fibers. Statistical analyses were performed for each speed category using One-Way Analysis of Variance (ANOVA) followed by a post hoc Tukey test. Data are presented as mean ± standard deviation (mean ± SD). Groups sharing the same letter indicate no statistically significant difference, whereas groups labeled with different letters indicate a statistically significant difference (*p* < 0.05), based on the post hoc test results.

Continuous NCVD curves revealed that Vin administration markedly reduced the proportion of fast-conducting fibers while shifting the distribution toward slower-conducting populations. In the quantitative evaluation based on subcategories of fiber groups, a significant increase in the relative proportion of slow-conducting fibers (<25 m/s) and a marked decrease in the proportion of fast-conducting fibers (>45 m/s) were observed in the Vin group. Injections of ALC or MitoQ alone did not alter fiber distribution profiles relative to the control group. Conversely, therapeutic co-treatments (Vin+ALC and Vin+MitoQ) partially reversed the fiber distribution shifts induced by Vin. There was no statistically significant difference among the groups in the proportion of fibers with moderate conduction velocity (25–45 m/s). Although both treatment agents showed a trend toward partial improvement in the fast fiber category, they remained statistically similar to the Vin group. In contrast, it was determined that the Vin+MitoQ group did not show any statistically significant differences compared to either the Con or Vin groups in the slow fiber category. Both treatment options led to partial improvements in fiber distribution, with neither treatment group showing a statistically significant difference from the other in slow- or fast-conducting fiber proportions (Figure 3).

**Figure 4:**
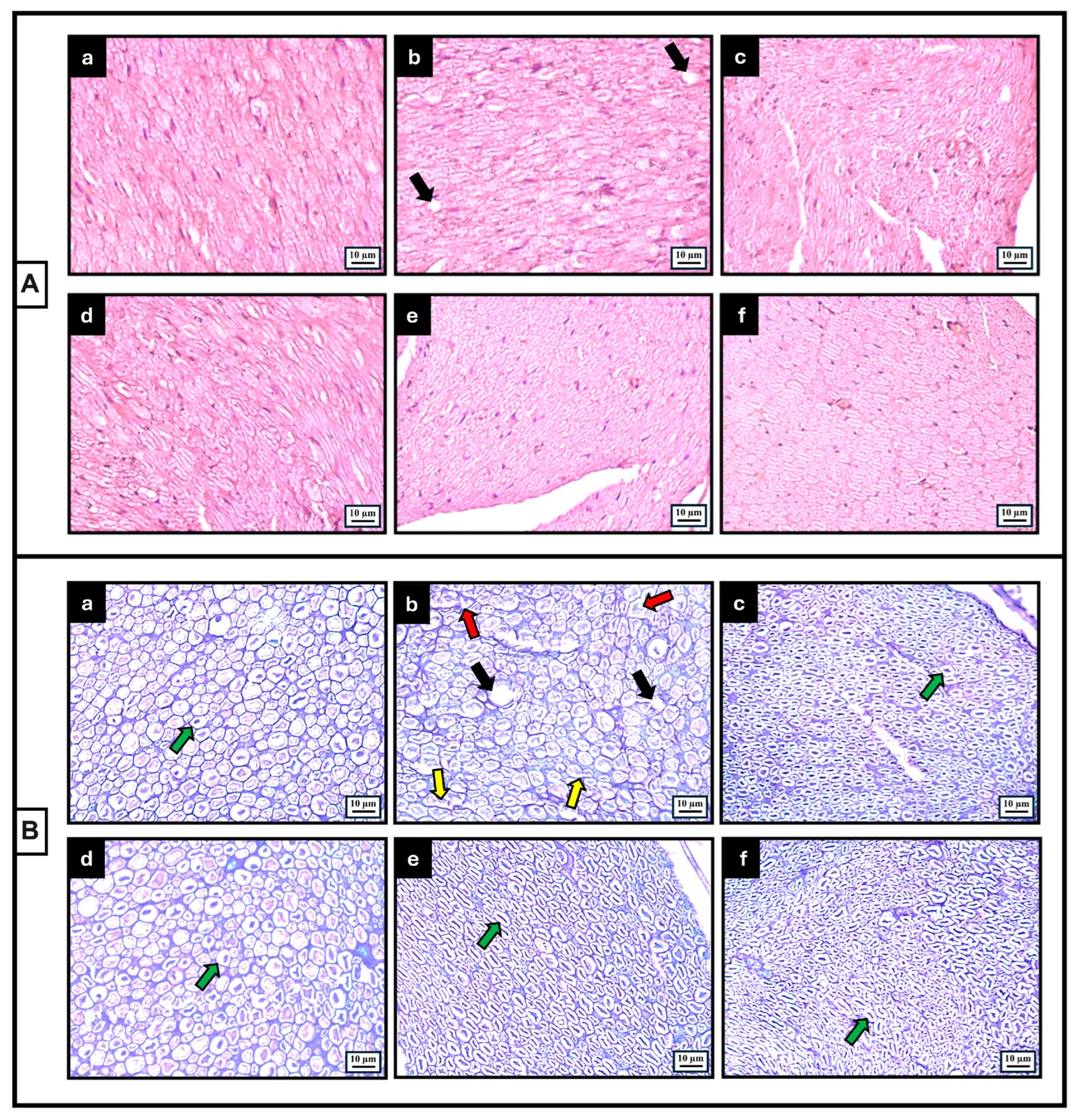
Representative light microscopy images of transverse sections of the sciatic nerve obtained from all experimental groups: Con (**a**), Vin (**b**), ALC (**c**), MitoQ (**d**), Vin+ALC (**e**), and Vin+MitoQ (**f**). (**A**) Hematoxylin and Eosin (H&E) staining showing general tissue architecture and axonal arrangement. (**B**) Klüver–Barrera (KB, Luxol Fast Blue + Cresyl Violet) staining demonstrating myelin sheath integrity and structural organization across groups. Structural features and pathological alterations across the experimental groups are indicated: green arrows denote normal myelinated nerve fibers exhibiting intact myelin sheaths and preserved axonal integrity, black arrows denote endoneurial vacuolization, yellow arrows indicate myelin sheath damage/splitting, and red arrows highlight axonal shrinkage/degeneration. Images were acquired at 40× objective magnification; scale bars represent 10 µm.

Histopathological examination of sciatic nerve sections from the control group using H&E staining revealed that the axonal structures and connective tissue organization exhibited a regular morphology (Figure 4A). In KB-stained sections, myelin sheaths formed regular, homogeneous, and continuous rings around axons, with no evidence of lamellar splitting or widespread myelin loss (Figure 4B). In contrast, severe neurotoxic damage parameters were detected in the group treated with Vin. In H&E staining, compared to the control group, localized atrophy of axonal structures and disruptions in fiber organization were notable, while minimal edema and vacuolar changes were observed in the interstitial spaces (Figure 4A). KB staining revealed widespread disruptions in myelin sheath integrity and irregularities in myelin rings, confirming Vin neurotoxicity (Figure 4B). In the ALC and MitoQ groups, where only protective agents were administered, tissue morphology was found to resemble that of the control group. Axonal organization and myelin density were preserved in both groups, indicating that these agents alone did not induce any major toxicity in peripheral nerve tissue (Figure 4A,B). In both the Vin+ALC and Vin+MitoQ cohorts, treatment significantly mitigated axonal disorganization, tissue disruption, and endoneurial vacuolization (Figure 4A). Furthermore, KB-stained sections demonstrated marked and comparable structural preservation of myelin sheath integrity following both antioxidant regimens (Figure 4B).

**Figure 5:**
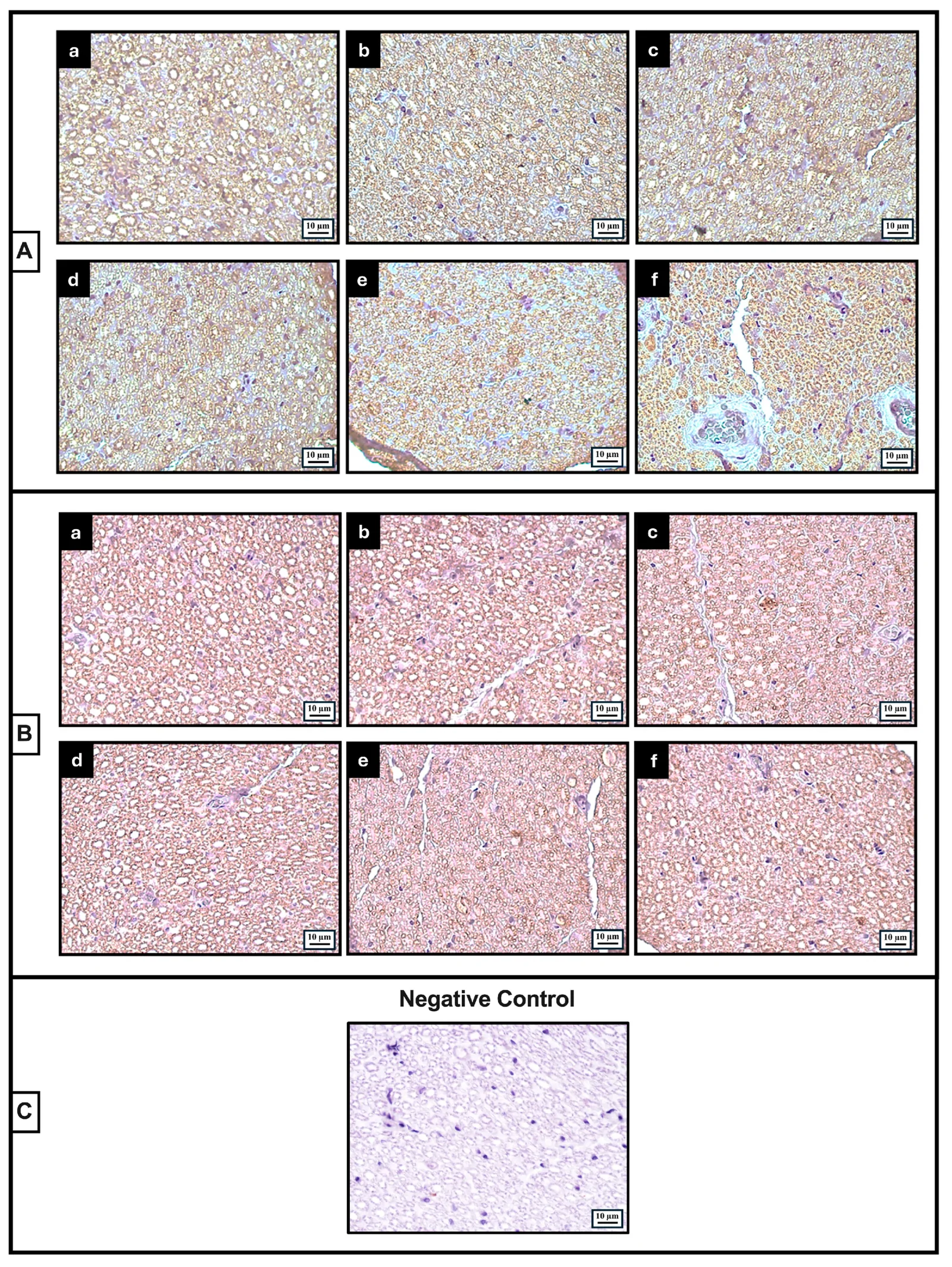
Representative immunohistochemical images of sciatic nerve transverse sections obtained from all experimental groups: Con (**a**), Vin (**b**), ALC (**c**), MitoQ (**d**), Vin+ALC (**e**), and Vin+MitoQ (**f**). (**A**) Myelin Basic Protein (MBP) immunoreactivity showing the distribution and density of myelin-specific proteins across groups. (**B**) Myelin Protein Zero (MPZ) immunoreactivity reflecting the structural organization of peripheral compact myelin. (**C**) Negative control section prepared by omitting the primary antibody to confirm staining specificity. Images were acquired at 40× objective magnification; scale bars represent 10 µm.

Control sections exhibited dense, continuous DAB-positive immunoreactive rings encircling axons for both MBP and MPZ, showing homogeneous distribution across the endoneurium. In contrast, microscopic examination of the stained preparations in the Vin-treated group revealed that the positive staining rings were fragmented in places, losing their continuity. In the ALC and MitoQ groups, where only protective agents were administered, uninterrupted MBP and MPZ expression was observed, and the ring morphology was found to be preserved. In contrast, the structural continuity of these immunoreactive rings was largely preserved in both the Vin+ALC and Vin+MitoQ groups (Figure 5A,B). No specific immunoreactive staining was observed in the negative control sections where the primary antibody was not applied. This finding confirms the specificity of the immunohistochemical staining (Figure 5C).

**Figure 6:**
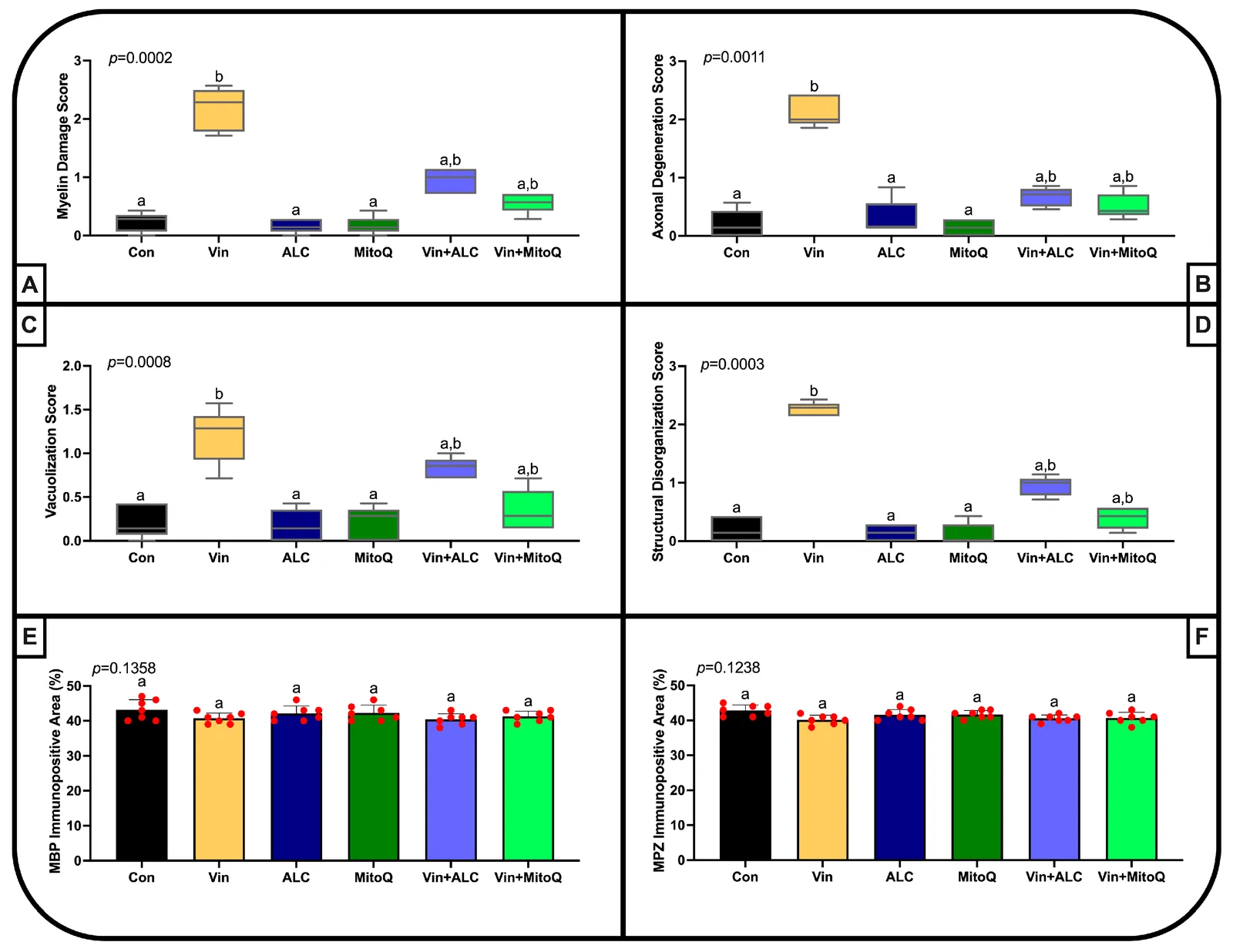
Histopathological scores and results of quantitative immunohistochemical analysis. (**A**–**D**) Statistical analysis of the semi-quantitative scoring data ((**A**): Myelin damage, (**B**): Axonal degeneration, (**C**): Vacuolization, (**D**): Structural disorganization) was performed using the Kruskal–Wallis test followed by a post hoc Dunn test. These data are presented as median (Q1, Q3). (**E**,**F**) Statistical analysis of continuous data ((**E**): %MBP-immunopositive area, (**F**): %MPZ-immunopositive area) was performed using one-way analysis of variance (One-Way ANOVA), followed by a post hoc Tukey test. These data are presented as mean ± standard deviation (mean ± SD). Groups sharing the same letter indicate no statistically significant difference, whereas groups labeled with different letters indicate a statistically significant difference (*p* < 0.05), based on the post hoc test results.

In histopathological evaluations of sciatic nerve sections, a significant increase in scores for myelin sheath damage, axonal degeneration, vacuolization, and structural disorganization was observed in the Vin group compared to the control group. It was determined that injections of ALC alone and MitoQ alone administered to healthy animals did not cause any damage to nerve tissue and remained at baseline levels similar to those of the Con group. Conversely, therapeutic intervention with ALC or MitoQ substantially attenuated all histopathological lesion scores, resulting in intermediate values that did not differ significantly from control levels. When median scores are evaluated, both Vin+ALC and Vin+MitoQ led to significant reductions in damage scores compared to the Vin group, though both co-treatment groups exhibited intermediate values overlapping with the control and injury groups (a,b designations). On the other hand, no statistically significant difference was detected between the groups in the percentages of MBP and MPZ immunopositive areas in the tissues. These quantitative findings demonstrate that, although Vin administration caused histopathological damage to the myelin sheath and axonal organization, it did not result in a significant loss in the total amount of major myelin proteins (MBP and MPZ) at the tissue section level (Figure 6).

## 4. Discussion

The present study comparatively evaluated the neuroprotective efficacy of sequential post-injury administration of ALC and the mitochondria-targeted antioxidant MitoQ in a rat model of Vin-induced peripheral neuropathy. The electrophysiological data we obtained demonstrate functional impairments in the electrical activity of the sciatic nerve following Vin administration. Parallel histopathological analyses confirmed marked structural damage induced by Vin, characterized by compromised myelin sheath integrity, axonal disorganization, and endoneurial vacuolization. Our findings demonstrate that ALC and MitoQ treatments—initiated following Vin-induced neurotoxicity—exhibit notable therapeutic efficacy in preserving peripheral nerve structural integrity and promoting functional recovery of electrophysiological parameters.

The cumulative Vin protocol adapted from Rahn et al. in 2007 (0.1 mg/kg/day administered in two 5-day cycles separated by a 2-day interval; cumulative dose: 1.0 mg/kg) reliably established peripheral neuropathy without systemic mortality [24]. Khalilzadeh et al. (2018) [33] emphasize that a cumulative Vin dose of 1.2 mg/kg, administered using a protocol similar to ours, also induced PN without causing mortality. However, in the same study, they noted that this treatment caused a significant decrease in body weight in the Vin group, contrary to our findings. This disparity may be largely attributed to differences in baseline animal age and weight; while younger, rapidly growing rats are more susceptible to chemotherapy-induced weight loss, the adult female rats (~330–350 g) used in our study had already reached a weight stabilization plateau. In line with this, Li et al. (2021) similarly reported no significant intergroup weight differences following comparable protocols [34]. Overall, this cumulative dosing regimen reliably induced robust peripheral neurotoxicity while maintaining overall animal viability, providing an optimal experimental model.

The antioxidant effects of the ALC and MitoQ treatment doses used in our study, as well as their ability to preserve mitochondrial bioenergetics, are based on previous studies that have confirmed these findings [25,26]. Patel et al. (2012) found that even a single administration of ALC at the same dose as ours following spinal cord injury was sufficient to reverse mitochondrial dysfunction [35]. In their study on traumatic brain injury, Zhou et al. (2018) discovered that a single 2 mg/kg dose of MitoQ treated traumatic injury through its antioxidant, neuroprotective, and anti-apoptotic effects, as well as by suppressing lipid peroxidation [36]. In their study on oxidative damage, Ibrahim et al. (2023) found that the exact same MitoQ administration protocol used in our study suppressed inflammatory responses such as TNF-α, IL-18, and IL-1β and restored the stabilization of mitochondrial membrane potential [37]. While these studies do not replace direct tissue-specific biochemical assays in our model, they provide a sound biological rationale for the therapeutic dosages selected and support the plausibility of mitochondrial involvement in the observed functional recovery.

Here, ex vivo electrophysiological recordings isolated the intrinsic electrical properties of the sciatic nerve trunk, eliminating systemic confounders associated with neuromuscular transmission or whole motor unit responses. Furthermore, the NCVD analyses conducted provide the opportunity to determine which characteristics of the fiber populations are associated with abnormalities in key CAP parameters [38]. Consequently, our findings provide novel insights into the comparative neuroprotective profiles of mitochondria-targeted versus conventional antioxidant strategies in Vin-induced neuropathy.

According to the results of our electrophysiological evaluation, Vin administration caused abnormalities in the CAP integral, amplitude, and overall waveform. Furthermore, NCVD analyses demonstrated a marked increase in the proportion of slow-conducting fibers (<25 m/s) following Vin exposure, which was substantially normalized toward baseline levels by MitoQ intervention. Furthermore, although not statistically significant, a trend toward partial improvement was observed in the fast-conducting fiber groups (>45 m/s) with MitoQ treatment. These electrophysiological alterations parallel previous observations in rabbit peripheral nerves, where Vin neurotoxicity was linked to microtubule disruption, secondary mitochondrial impairment, and axonal degeneration [39]. While ALC primarily supports mitochondrial lipid transport and metabolic recovery [15], Chen et al. [23] demonstrated that MitoQ alleviates Vin-induced neuropathic pain via Nrf2 pathway activation and mitochondrial preservation. Furthermore, numerous studies emphasize that MitoQ effectively treats impaired mitochondrial energy production and metabolism [40]. Peripheral nerve regeneration involves energy-intensive processes such as the proliferation of Schwann cells and the synthesis of new membrane and cytoskeletal structures; the sustainability of these processes depends largely on mitochondrial ATP production [41]. In this context, when our electrophysiological and structural responses are evaluated as a whole, MitoQ offers a favorable recovery trend compared to ALC in preserving nerve function and morphology. It should be emphasized that while MitoQ’s targeted mitochondrial profile provides a strong biological rationale, the observed functional and structural recovery cannot be attributed solely to a single bioenergetic mechanism. Given the multi-faceted nature of Vin neurotoxicity, several complementary pathways may have contributed to these therapeutic outcomes. These include non-specific ROS scavenging, attenuation of secondary inflammatory cascades, preservation of Schwann cell viability, and maintenance of axonal membrane stability. Furthermore, pharmacokinetic and bioavailability differences between MitoQ and ALC may also account for the observed nuances in electrophysiological and histopathological recovery profiles.

The histopathological evaluations conducted in our study clearly demonstrate the cellular and morphological manifestations of electrophysiological function loss and recovery in the treatment groups. In their study, El-Sawaf et al. (2024) used the same Vin protocol as we did and, consistent with our findings, detected axonal degeneration, interstitial edema, and Schwann cell proliferation [42]. Similarly, Yang et al. (2023) identified neurotoxic damage associated with increased oxidative stress (elevated MDA and NO; decreased GSH, SOD, and CAT) in the sciatic nerves of rats treated with Vin and demonstrated that an antioxidant agent (L-theanine) significantly reduced this damage [14]. This supports the possible antioxidant mechanism underlying the histological protection observed in our ALC and MitoQ groups.

On the other hand, despite the marked myelin damage and vacuolization caused by the Vin treatment in sciatic nerve sections, no significant difference was observed between the groups in the total percentage of immunopositive areas for the major myelin proteins MBP and MPZ. It is known from the literature that the development of Vin-induced demyelination requires quite long exposure periods. Ja’afer et al. (2006) reported relative myelin loss even after a 14-week waiting period following 5 weeks of Vin administration [43]. Accordingly, the electrophysiological deficits observed in our model appear to be driven by structural disorganization, myelin lamellar loosening, and endoneurial vacuolization rather than overt demyelination or net myelin protein depletion.

This study focused on the potential therapeutic effects of ALC and MitoQ on possible impairments in Vin-induced electrophysiological responses in the rat sciatic nerve, as well as the underlying morphological changes associated with these effects. However, since only female rats were utilized in the present study to avoid gender-dependent baseline variations, these findings cannot be directly generalized to males or used to infer sex-specific therapeutic differences. Furthermore, apart from the immunohistochemical labeling of MBP and MPZ proteins, no measurements of molecular substance levels or gene expression levels were performed. In addition, direct isolated mitochondrial bioenergetic parameters—such as ATP production rates, mitochondrial membrane potential, and mtROS accumulation—as well as direct tissue accumulation of MitoQ were not quantified in the nerve tissue. Consequently, while our study demonstrates clear functional and morphological recovery, the exact molecular pathway driving this rescue remains to be fully delineated. Moreover, quantitative morphometric parameters such as the g-ratio were not assessed, as the standard paraffin-embedding protocol utilized here is prone to processing artifacts that preclude reliable high-resolution axonal measurements. Direct biochemical assays of tissue redox balance (such as MDA, SOD, or catalase activities) and ultrastructural evaluation of axonal cytoskeletons and mitochondrial cristae via transmission electron microscopy (TEM) were not conducted due to the methodological constraints of paraffin processing and animal welfare principles restricting post hoc cohort additions. While our study was specifically designed to isolate peripheral nerve biophysical properties and tissue structural integrity using terminal ex vivo methods, functional outcomes were not accompanied by in vivo longitudinal behavioral assays (such as mechanical allodynia, thermal hyperalgesia, motor coordination, or quantitative gait analysis) to directly evaluate sensory and motor neuropathic pain phenotypes. Additionally, these findings are restricted to ex vivo rodent preparations and warrant eventual validation in higher-order animal models or translational human cellular platforms. In our data, the post-treatment evaluation period is relatively short and focuses primarily on short-term functional and structural improvement. Although the sample size (*n* = 7 per group) provided sufficient statistical power to detect major neurotoxic and therapeutic treatment effects relative to injury, larger sample sizes may be required in future studies to detect subtle differences between active co-treatments. Nevertheless, our findings provide direct, quantifiable insights into peripheral nerve pathophysiology without systemic neuromuscular interference. Finally, a notable limitation of the present study is the use of healthy, non-tumor-bearing animal models. While this approach was chosen to isolate the specific neuroprotective and hemodynamic effects of MitoQ against Vin toxicity without systemic oncological confounders, it does not account for potential interactions with tumor biology, chemotherapy pharmacokinetics, or multi-agent regimens. Future translational investigations utilizing tumor-bearing models will be essential to verify whether MitoQ preserves Vin’s antitumor efficacy while maintaining its neuroprotective benefits.

## 5. Conclusions

In conclusion, vincristine, an essential chemotherapeutic agent in clinical oncology, frequently induces debilitating peripheral neuropathy that limits therapeutic dosing and impairs patients’ quality of life. Electrophysiological and histopathological analyses demonstrated that post-injury administration of either ALC or MitoQ exhibited substantial neuroprotective efficacy, significantly restoring sciatic nerve functional conductivity and structural architecture. Notably, the mitochondria-targeted profile of MitoQ conferred favorable recovery trends, particularly across myelin sheath integrity and compound action potential kinetics. These findings indicate that targeting mitochondrial oxidative stress and bioenergetic impairment represents a promising translational strategy for mitigating chemotherapy-induced neurotoxicity.

## Data Availability

The data presented in this study are available upon reasonable request from the corresponding author.

## Abbreviations

The following abbreviations are used in this manuscript:

ALC	Acetyl-L-carnitine
CAP	Compound action potential
H&E	Hematoxylin and Eosin
KB	Klüver–Barrera
LFB	Luxol fast blue
MBP	Myelin basic protein
MPZ	Myelin protein zero
NCVD	Nerve conduction velocity distribution
PN	Peripheral neuropathy
ROS	Reactive oxygen species
Vin	Vincristine
